# Prognostic Prediction Using Baseline Heart Rate and Head-Up Tilt Test Mode in Pediatric Cardioinhibitory Vasovagal Syncope with Non-Pharmacological Therapy

**DOI:** 10.3390/jcm15166169

**Published:** 2026-08-09

**Authors:** Shuo Wang, Yuwen Wang, Fang Li, Xuemei Luo, Hong Cai, Runmei Zou, Cheng Wang

**Affiliations:** Department of Pediatric Cardiovasology, Children’s Medical Center, The Second Xiangya Hospital, Central South University, Changsha 410011, China; wangshuo998@sina.com (S.W.); wangyw@csu.edu.cn (Y.W.); lifang1024@csu.edu.cn (F.L.); luoxuemei@csu.edu.cn (X.L.); ralise@csu.edu.cn (H.C.); zourm0721@csu.edu.cn (R.Z.)

**Keywords:** head-up tilt test, cardioinhibitory response, vasovagal syncope, prognosis, children

## Abstract

**Objectives:** Clinically feasible simple methods for prognostic assessment of cardioinhibitory vasovagal syncope (VVS) are rare. This study investigated the predictive utility of head-up tile test (HUTT) data among cardioinhibitory VVS children with non-pharmacological therapy. **Methods:** A retrospective analysis was conducted on the clinical data of 403 children with VVS who had cardioinhibitory responses induced by HUTT. The children were aged 4–18 years old, including 178 males and 225 females. After the non-pharmacological interventions and follow-up, they were divided into the good prognosis group (233 cases) and the poor prognosis group (170 cases). **Results:** (1) Comparison between groups: Compared with the good prognosis group, the baseline heart rate (HR0) in the poor prognosis group was lower, and the proportion of sublingual nitroglycerin-provoked HUTT (SNHUT) was higher (*p* < 0.05). (2) Univariate analysis: Univariate analysis showed that HR0 was a protective factor on the prognosis of VVS, while SNHUT was a risk factor for the prognosis of VVS. (3) Multivariate analysis: HR0 was an independent protective factor on the prognosis of VVS. That is, for every 1 bpm increase in HR0, the risk of poor prognosis of VVS decreased by 3%. SNHUT was an independent risk factor for the prognosis of VVS, and the risk of poor prognosis of VVS increased by 3.34 times compared with basic HUTT (BHUT). (4) Evaluation of diagnostic tests: The combination of HR0 and HUTT mode had a good prognostic prediction effect for VVS (AUC = 0.71, *p* < 0.001). **Conclusions:** The combination of HR0 and HUTT mode demonstrates robust prognostic predictive value for non-pharmacological interventions in pediatric patients with VVS exhibiting cardioinhibitory responses during HUTT.

## 1. Introduction

Head-up tilt test (HUTT) can induce or reproduce clinical syncope symptoms and has become an effective tool for diagnosing neurally mediated syncope (NMS) in clinical practice [1]. Vasovagal syncope (VVS) is the most common subtype of pediatric NMS encountered clinically. It can be categorized into three hemodynamic subtypes according to response patterns during tilt testing: vasoinhibitory type (VVS-VI), cardioinhibitory type (VVS-CI), and mixed type (VVS-M). Among these subtypes, both VVS-CI and VVS-M manifest cardioinhibitory responses characterized by heart rate (HR) reduction when provoked by HUTT [2,3]. VVS complicated with cardiac arrest lasting longer than 3 s is defined as malignant VVS [4,5,6].

The existing literature has demonstrated that the overall prognosis of patients with VVS is considerably more favorable compared with individuals suffering from unexplained syncope and cardiogenic syncope [7]. The underlying mechanism is thought to involve enhanced myocardial contraction and subsequent activation of left ventricular mechanoreceptors during the syncope cascade [8]. Although NMS is generally regarded as a benign clinical entity, long-term close follow-up data remain limited. Severe syncope triggered by HUTT may cause marked declines in HR or blood pressure (BP), which can further provoke convulsions or seizure-like manifestations in susceptible children [9,10].

Previous evidence suggests that HUTT data facilitate early recognition of pediatric malignant VVS induced by HUTT [11]. Nevertheless, studies focusing on prognostic stratification for children with cardioinhibitory VVS remain scarce. In the present study, we retrospectively collected clinical data from pediatric patients presenting with HUTT-evoked cardioinhibitory responses accompanied by HR reduction over a 20-year period at a single center. We aimed to explore predictive indicators for the effectiveness of clinical non-pharmacological interventions.

## 2. Methods

### 2.1. Study Population

A retrospective analysis was conducted on the clinical data of 403 children with VVS who had a decrease in HR due to cardioinhibitory responses induced by the HUTT and were followed up for their prognosis after non-pharmacological intervention. These children were treated in the Department of Pediatric Cardiovascular Diseases of The Second Xiangya Hospital, Central South University, from November 2002 to January 2022 due to unexplained syncope or presyncope symptoms (such as dizziness, headache, chest pain, chest tightness, sighing, amaurosis, etc.). After detailed medical history inquiries, careful physical examinations, electrocardiograms (ECG), Holter ECG, cardiac X-ray, echocardiograms, electroencephalograms, cranial CT or MRI, and blood biochemical examinations (including fasting blood glucose and myocardial enzymes), organic heart, brain, lung, vascular diseases, immune diseases, and psychogenic diseases were excluded. However, the causes of their syncope or presyncope symptoms remained unclear. The HUTT was then performed after obtaining the written informed consent of the patients or/and their family members.

A total of 820 cardioinhibitory VVS patients were enrolled. After excluding those who were lost to follow-up or had poor compliance with the non-pharmacological treatment, 403 cases were finally included in the study. They were aged between 4 and 18 years old, including 178 males and 225 females.

**Figure 1:**
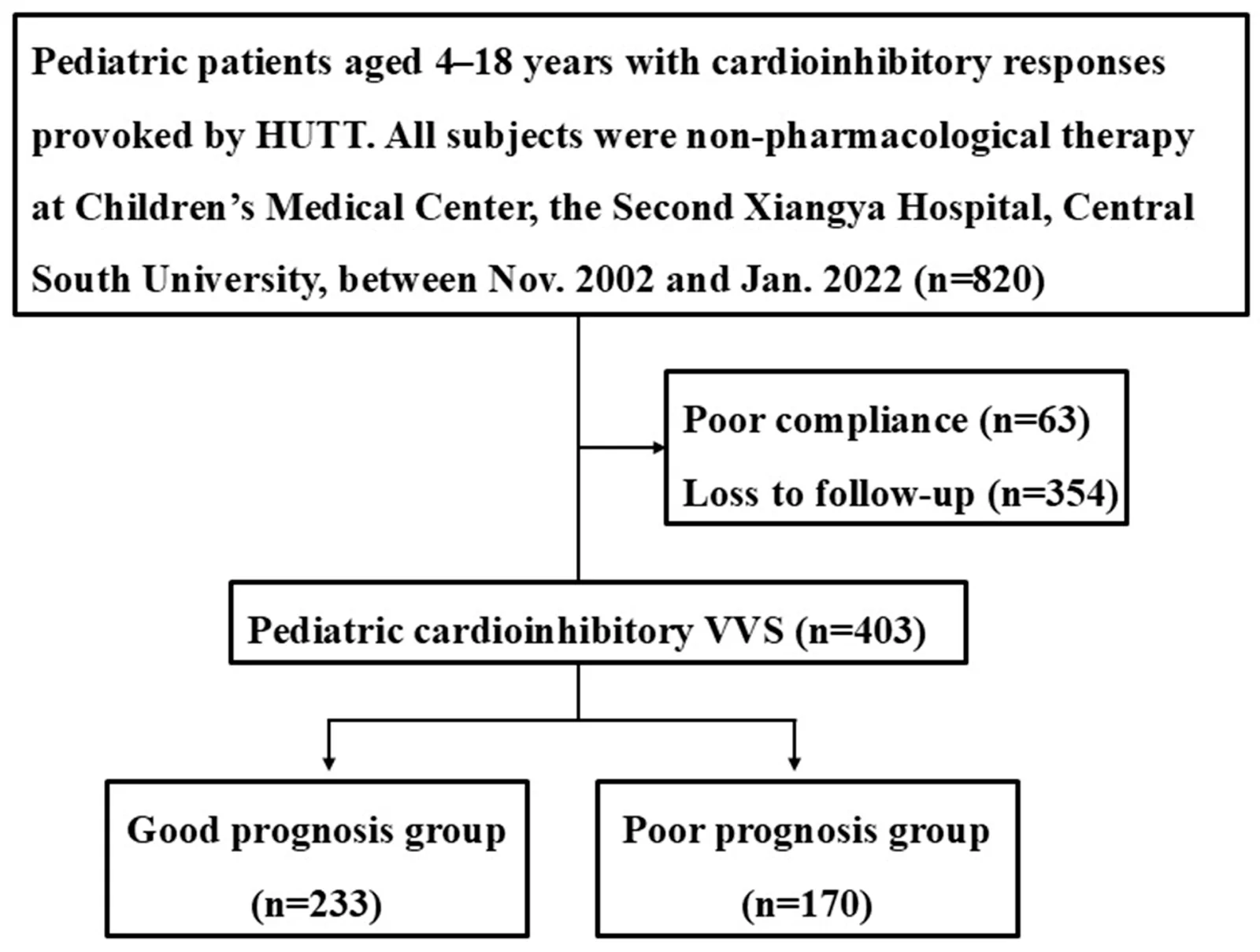
Flow chart.

All cardioinhibitory VVS patients underwent a standardized three-month course of non-pharmacological treatment (including health education, upright training, increased water and salt intake); HUTT was repeated and symptoms were reassessed. Based on whether their symptoms and HUTT results continued to meet the criteria for cardioinhibitory VVS, the cohort was divided into a good prognosis group (233 cases) and a poor prognosis group (170 cases) (Figure 1).

The research was conducted according to the guidelines of the Declaration of Helsinki and approved by The Second Xiangya Hospital, Central South University [Ethical Audit No. Study 249 (2022)].

### 2.2. Head-Up Tilt Test

#### 2.2.1. Preparation Before the Test

Subjects were required to discontinue cardiovascular active drugs for more than 5 half-lives before the test and avoid consuming foods that might affect autonomic nerve function, such as coffee. They were also asked to fast and refrain from drinking for 4 h before the test. The subjects or their guardians were informed about the precautions before the test and the possible risks during the test process, and the written informed consent was signed by the subjects themselves or their legal guardians. The HUTT has two modes: the basic HUTT (BHUT) and the sublingual nitroglycerin-provoked HUTT (SNHUT) [2].

#### 2.2.2. Basic Head-Up Tilt Test

The tilt devices used were the Head-up Tilt Test System (ST-711) of Beijing Juchi Medical Technology Co., Ltd., Beijing, China, and the Head-up Tilt Test Monitoring System (SHUT-100) of Jiangsu Standard Medical Technology Co., Ltd., Wuxi, China. The examination was scheduled between 8:00 and 11:00 in the morning. The room temperature was maintained at 20–24 °C. In a quiet environment, the subjects emptied their bladders, then lay on the tilt diagnostic bed in a supine position and rested for 10 min. Their chests and knee joints were fixed with straps. The baseline HR, BP, and ECG were recorded. Then, within 15 s, the position was changed to a head-up and feet-down tilt of 60°. The clinical manifestations, HR, BP, and ECG, were continuously monitored until a positive reaction occurred, at which point the test was terminated and the supine position was restored within 10 s. If any discomfort symptoms appeared, monitoring would be carried out at any time until a positive reaction occurred or the 45 min time limit specified in the detection procedure was reached.

#### 2.2.3. Sublingual Nitroglycerin-Provoked Head-Up Tilt Test

For those who had a negative result in the BHUT, they continued to maintain the same tilted position. The subjects were given sublingual nitroglycerin tablets at a dose of (4–6) μg/kg (with the maximum dose not exceeding 300 μg). The clinical manifestations, HR, BP, and ECG, were continuously monitored until a positive reaction occurred or until 20 min had passed without a positive reaction, in which case the test was terminated. Then, the tilt diagnostic bed was quickly returned to the horizontal position, and the clinical manifestations, HR, BP, and ECG, were continuously monitored in the supine position until these indicators returned to normal.

#### 2.2.4. Diagnostic Criteria for Vasovagal Syncope

Syncopal episodes or presyncope symptoms together with any of the following responses in the HUTT are considered positive responses: (1) systolic BP (SBP) ≤80 mmHg (1 mmHg = 0.133 kPa) or diastolic BP (DBP) ≤50 mmHg or mean BP decrease ≥25%; (2) HR <75 bpm for 4 to 6 years younger children, <65 bpm for >6 to 8 years old children, and <60 bpm for those >8 years old children and adolescents; (3) ECG showing sinus arrest or junctional escape rhythm; and (4) atrioventricular block (II° or III°) or cardiac arrest ≥3 s.

VVS hemodynamic type: (1) VVS-VI: predominantly a decrease in BP with no significant change in HR. (2) VVS-CI: predominantly a decrease in HR with no significant change in BP. (3) VVS-M: a decrease in both HR and BP [2].

### 2.3. Variables

Age, sex, height, weight, body mass index (BMI), the syncope times in medical history, HR and BP (SBP, DBP) at baseline (HR0, SBP0, DBP0) and when syncope (HRs, SBPs, DBPs) was induced by the HUTT, and the mode of HUTT (BHUT, SNHUT).

### 2.4. Intervention Measures

(1) Health education: Avoid triggering factors such as standing for a long time, sudden changes in body position, and crowded or stuffy environments, and avoid anxiety. (2) Orthostatic training: Stand with the back of the head and the upper part of the back against the wall, with the heels of both feet 15 cm away from the wall. The duration of standing is based on the time that the child can tolerate. Generally, it starts from 5 min each time, twice a day, and gradually increases to 30 min each time. (3) Increase water and salt intake: Drink 30–50 mL/kg of water daily to keep the urine clear. Oral rehydration salts III can be given in combination (5.125 g each time, dissolved in 250 mL of warm water, taken orally in divided doses, twice a day. The dose for children under 6 years old is halved). Oral rehydration salts III are produced by Xi’an Anjian Pharmaceutical Co., Ltd. (Xi’an, China) (Approval number: H20090205; specification: 5.125 g/bag; Formula: 3.375 g Anhydrous glucose, 0.65 g Sodium chloride, 0.375 g Potassium chloride, and 0.725 g Sodium citrate; Osmotic pressure: 245 mOsm/L; Sodium salt concentration: 75 mmol/L; Tonicity: 1/2) [2,12].

### 2.5. Follow-Up and Prognosis Judgment

Patients returned to the hospital for follow-up 90 days after the initial diagnosis and intervention. The presence or absence of symptoms of syncope or presyncope in the child was assessed to determine if they had disappeared, improved, or worsened, and the HUTT was repeated. (1) Subjective manifestations: The symptoms of syncope or presyncope disappear, improve, or worsen when the patient is followed up. (2) Objective manifestations: Good prognosis: When the HUTT is repeated, the time for inducing syncope is prolonged compared with that at the first diagnosis, or the result changes from positive in BHUT to positive in SNHUT at the first diagnosis, or from positive in HUTT to negative. Poor prognosis: When the HUTT is repeated, the time for inducing syncope is shortened compared with that at the first diagnosis, or the result changes from positive in SNHUT to positive in BHUT, or from negative in HUTT to positive in HUTT [13,14].

### 2.6. Statistical Analysis

Continuous variables with a normal distribution are presented as mean ± SD ($\bar{x}$ ± *s*) and were compared between groups using Student’s *t*-test. Non-normally distributed data are presented as median (interquartile range) and were compared using the Mann–Whitney U-test. Categorical variables are presented as numbers (percentages) and were compared using the Chi-square test or Fisher’s exact test. Univariate regression is used to explore potential associations between factors and outcomes, while multivariate regression is used to verify the independence of the influencing factors on the outcome. Regression equations are used to identify the final independent factors that influence the outcome. Model performance is evaluated using internal cross-validation and through receiver operating characteristic (ROC) curves, calibration curves, and decision curve analysis (DCA). Finally, a nomogram is plotted to facilitate clinical application. All the analyses were performed with the statistical software Packages R (version 3.6.1) (http://www.R-project.org, The R Foundation, Vienna, Austria) and EmpowerStats (http://www.empowerstats.com, X & Y Solutions, Inc, Boston, MA, USA). *p*-values < 0.05 (two-sided) were considered statistically significant.

## 3. Results

### 3.1. Comparison Between Groups

There were statistically significant differences in HR0 and HUTT modes between the good prognosis group and the poor prognosis group. Compared with the good prognosis group, the HR0 in the poor prognosis group was lower, and the proportion of SNHUT was higher (*p* < 0.05) (Table 1).

### 3.2. Univariate Analysis

The univariate analysis showed that HR0 was a potential protective factor on the prognosis of VVS. For every 1 bpm increase in HR0, the risk of poor prognosis of VVS decreased by 2%. SNHUT was a potential risk factor for the prognosis of VVS. Compared with BHUT, the risk of poor prognosis in SNHUT increased by 4.26 times. For VVS-CI and VVS-M compared with VVS-VI, the trend of the risk of poor prognosis of VVS was smaller, and the prognosis of the VVS-CI was better than that of the VVS-M. The *p*-value was close to being significant, which might be limited by the influence of sample size. Similarly, the HRs might also be a risk factor for the prognosis of VVS (Table 2).

### 3.3. Prediction Model for the Prognosis of VVS

After adjusting for demographic factors, the protective effect of HR0 on the prognosis of VVS remained unchanged compared with the univariate result (the odds ratio value remained unchanged, *p* < 0.05). When other relevant factors were continuously added for a full model adjustment, the protective effect of HR0 on the prognosis of VVS was still significant and stable (the fluctuation of the OR value was less than 10%, *p* < 0.05). This indicated that HR0 had an independent protective effect on the prognosis of VVS. That is, for every 1 bpm increase in HR0, the risk of poor prognosis of VVS decreased by 3%. However, after adjusting for demographic factors, compared with the univariate result, the hazardous effect of SNHUT on the prognosis of VVS compared with BHUT was increased, and the effect was still significant (the fluctuation of the OR value was less than 10%, *p* < 0.05). When other relevant factors were continuously added for a full model adjustment, the hazardous effect of SNHUT on the prognosis of VVS compared with BHUT was decreased (*p* < 0.05). This indicated that SNHUT compared with BHUT was an independent risk factor for the prognosis of VVS. That is, compared with BHUT, the risk of poor prognosis of VVS in SNHUT increased by 3.34 times (Table 3).

### 3.4. Evaluation of the Diagnostic Test for Predictive Values

To understand the comprehensive judgment effect of these two independent influencing factors on the prognosis of VVS, binary regression analysis was performed through the fitting of HR0 and HUTT modes.

**Figure 2:**
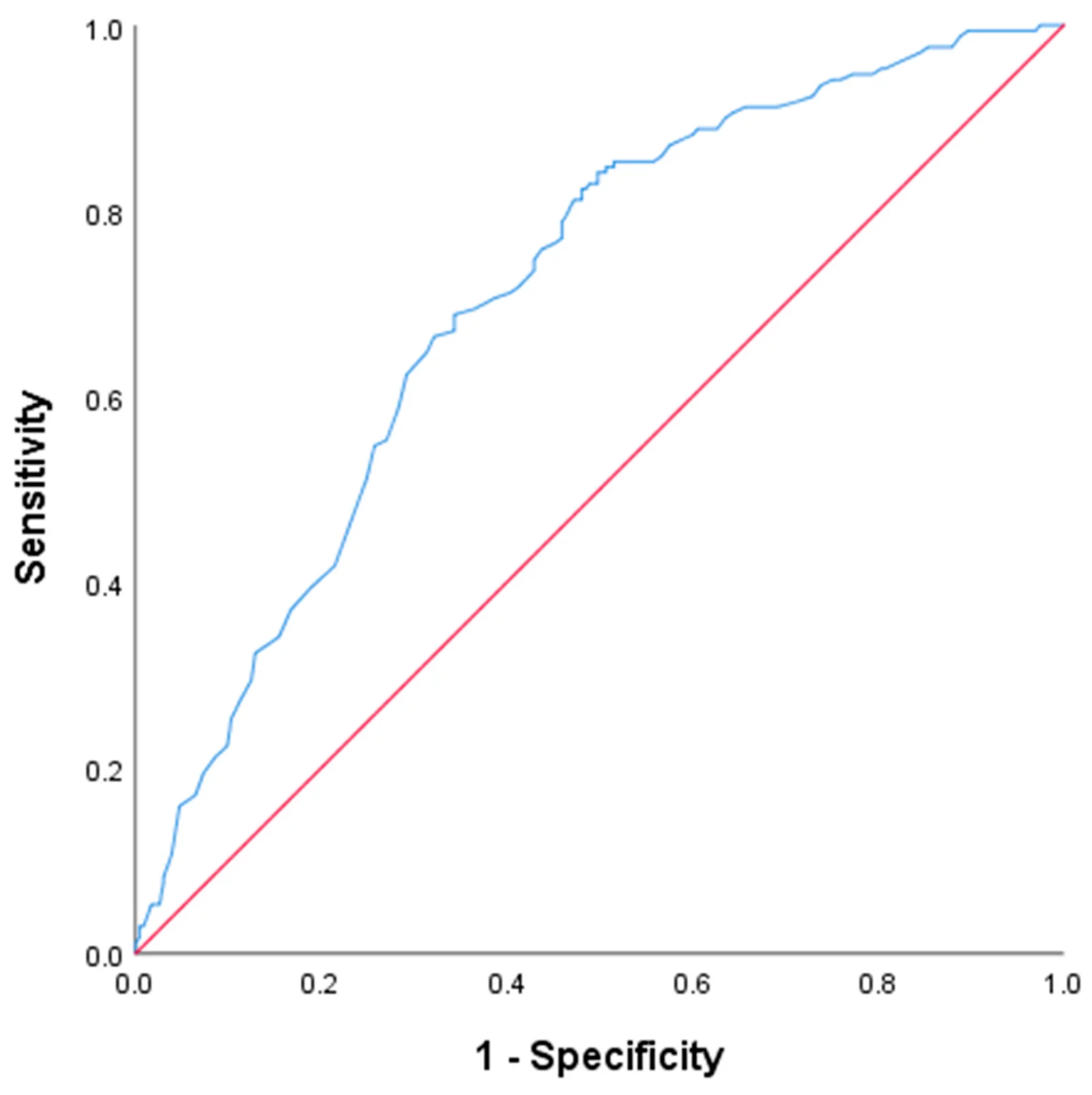
ROC of combined HR0 + HUTT mode indicators predicting poor prognosis of VVS.

An ROC curve analysis of combined indicators for predicting poor prognosis in pediatric patients with cardioinhibitory VVS showed an AUC of 0.710, a sensitivity of 68.82%, and a specificity of 65.67% (Table 4 and Figure 2).

### 3.5. Evaluating Predictive Models Using Calibration Curves and DCA

**Figure 3:**
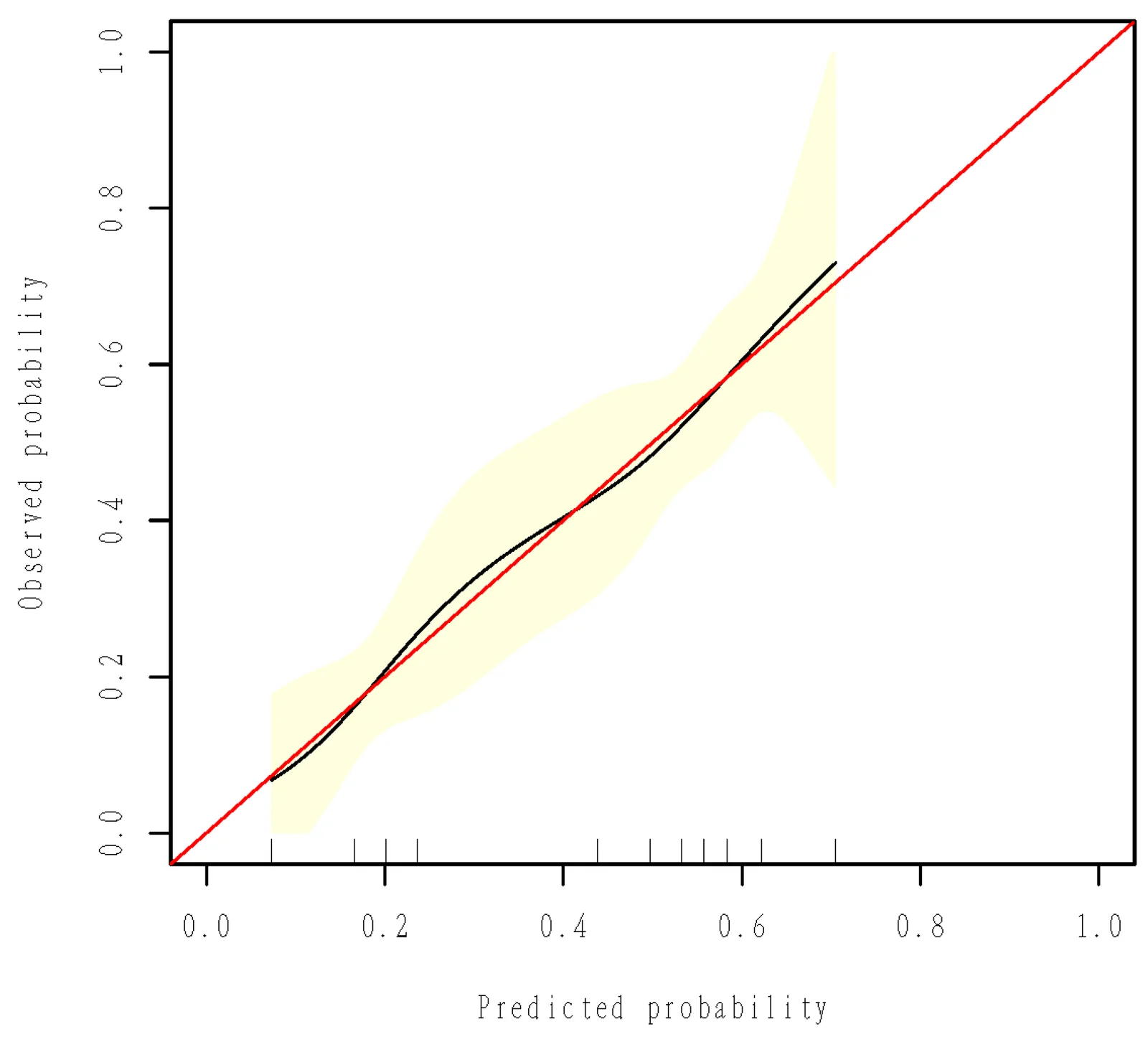
Calibration curve of the prediction model.

**Figure 4:**
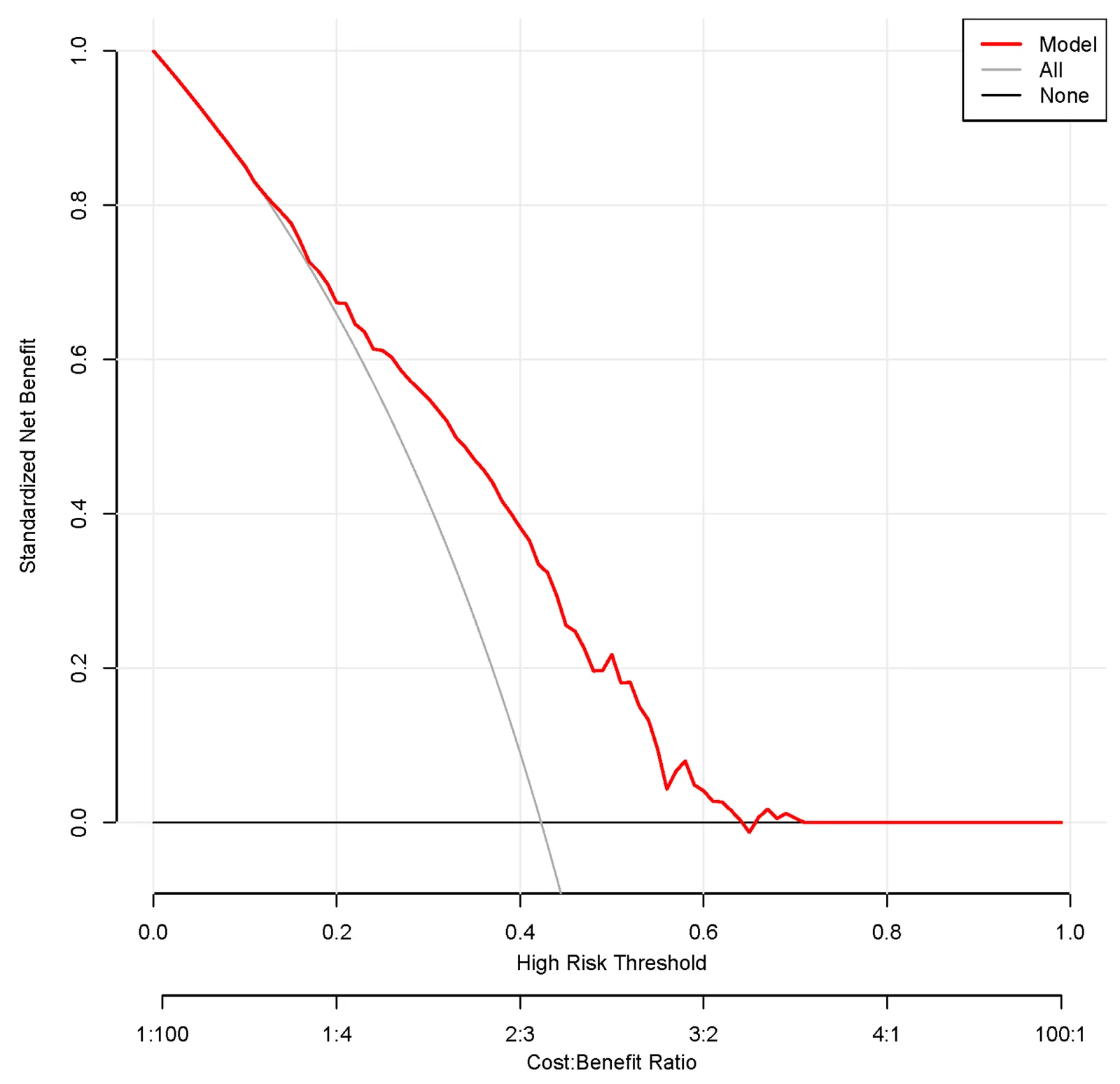
DCA of the prediction model.

The Hosmer–Lemeshow test indicates that the model has a good fit (*p* > 0.9). When the threshold probability ranged from 0.2 to 1.0, the net benefit of using the composite index to predict poor prognosis in children with cardioinhibited VVS ranged from 0.01 to 0.8, indicating that the net benefit of using the composite index to predict poor prognosis in these children was greater than that of directly determining a poor prognosis. Therefore, the threshold probability was set to >0.2 (Figure 3 and Figure 4).

### 3.6. Nomogram for Predictive Models

**Figure 5:**
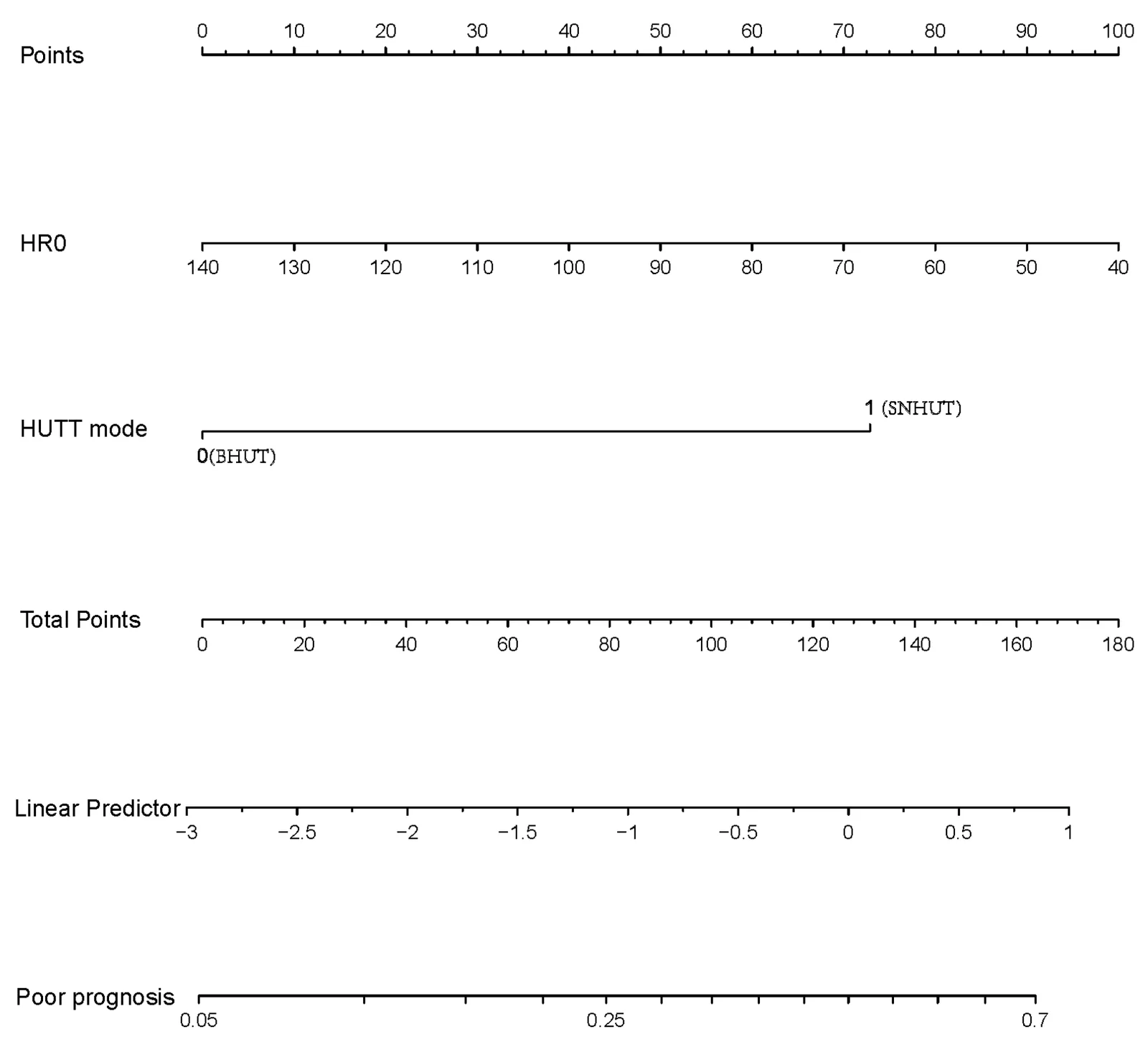
Nomogram of the prediction model.

Based on the multivariate Logistic regression analysis and model evaluation, scores were assigned to the data of HUTT mode (BHUT = 0, SNHUT = 1) and HR0 (bpm) respectively. The scores of each factor were added together to obtain the total score. The total score was then converted into a predicted probability, and a nomogram for predicting the prognosis of pediatric cardioinhibitory VVS was constructed (Figure 5). Different values of each variable included in the nomogram have their vertically corresponding scores on the top scoring scale. The scores of each item are added together to get the total score, and the corresponding point on the bottom probability line represents the probability of poor prognosis.

Logit (poor prognosis) = 0.30057 − 0.02306 × HR (bpm) + 1.68057 × HUTT mode (BHUT = 0, SNHUT = 1)

## 4. Discussion

Cardioinhibitory responses can be induced by HUTT provocation or excessive vagal hyperactivity, and marked HR deceleration during this process may trigger severe complications including convulsions and life-threatening adverse events. The present study retrospectively collected clinical data from pediatric patients over a 20-year period at a single center (2002–2022). Notably, all patient inclusion, indicator definition, prognostic outcome judgment, and data analysis in this study were uniformly performed in accordance with the latest unified clinical criteria and standardized diagnostic protocols at the time of manuscript writing, rather than adopting the historical clinical standards of each enrollment year [2]. This unified analytical strategy eliminates potential temporal heterogeneity caused by the iterative updating of HUTT operational specifications, clinical intervention schemes, and follow-up management protocols over the years, ensuring consistent and reliable outcome evaluation and prognostic stratification across the entire cohort. In our cohort of children with HUTT-induced cardioinhibitory VVS, patients with poor prognosis exhibited significantly lower HR0 and a higher proportion of SNHUT compared with those with favorable prognosis. Further statistical analysis confirmed that HR0 serves as an independent protective prognostic factor, whereas SNHUT acts as an independent risk factor for therapeutic outcomes.

HUTT-induced cardioinhibitory response is essentially a compensatory cardiac self-protective physiological mechanism. Clinically, VVS is characterized by abrupt hypotension and bradycardia, which result from sympathetic nervous suppression and vagal nerve overactivation, respectively [15]. The vasovagal reflex is widely recognized as an endogenous cardiac defense mechanism triggered by physical stress or potential danger. The reflex-mediated HR reduction favorably reduces myocardial oxygen consumption and facilitates ventricular diastolic filling and coronary perfusion. Vagus nerve-mediated atrioventricular block is paroxysmal, confined to the atrioventricular node, and closely correlated with sinus bradycardia, with most patients maintaining normal atrioventricular conduction; therefore, this type of block is generally benign and does not require pacemaker implantation in asymptomatic individuals [16]. Consistent with this viewpoint, Omar et al. [17] demonstrated that prolonged HUTT-induced cardiac arrest shows poor reproducibility, and permanent pacemaker implantation should not be regarded as first-line therapy for malignant VVS. van Lieshout et al. [18] further clarified the autonomic regulatory mechanism of vasovagal responses, illustrating that bradycardia originates from excessive efferent vagal activation, while hypotension is attributed to suppressed sympathetic activity and reduced arterial vascular resistance.

Two distinct neural pathways mediate vasovagal reflexes, namely central and peripheral pathways. The central-type response is triggered by emotional stress or pain via direct hypothalamic activation of the medullary cardiovascular center. In contrast, the peripheral-type response arises from reduced central blood volume and enhanced cardiac contractility, which collectively stimulate ventricular mechanoreceptors to induce vasodilation and bradycardia. Afferent signals from cardiac and vascular stretch receptors may produce contradictory reflex effects. The peripheral inhibitory vasovagal reflex overrides the physiological baroreflex loop, leading to imbalanced volume–pressure regulation in the cardiovascular system. Accordingly, vasovagal responses are not pathological in essence; the underlying neural pathways exist in healthy populations, and individual susceptibility differences primarily account for the occurrence of syncope episodes. For pediatric VVS management, non-pharmacological intervention is universally recommended as the first-line therapeutic strategy [2,12]. Reybrouck et al. [19] verified that continuous tilt training effectively corrects abnormal autonomic reflexes and benefits both VVS-VI and VVS-CI patients. In contrast, pharmacological treatment yields limited clinical efficacy for VVS [20]. For patients with refractory VVS-CI unresponsive to non-pharmacological interventions, pacemaker implantation can be considered as an alternative remedy. However, the ISSUE-3 study reported by Brignole et al. [21] indicated that cardiac pacing effectively reduces syncope recurrence in patients with documented asystolic NMS, while its therapeutic benefit is insufficiently validated for HUTT-positive patients, even those with spontaneous cardiac arrest history. Consistent with clinical guidelines, the present study exclusively focused on non-pharmacological intervention for enrolled pediatric VVS patients.

VVS is generally considered a benign condition with a favorable long-term prognosis. A retrospective cohort study by Ayabe et al. [22] revealed a low syncope recurrence rate of 16.9% during long-term follow-up, with no significant prognostic difference between HUTT-positive and HUTT-negative patients. Similarly, Bałczewska et al. [23] confirmed excellent prognosis in syncope patients without structural cardiac lesions or electrocardiographic abnormalities, supporting lifestyle intervention as the core management strategy, including trigger avoidance, physical exercise, and enhanced water and salt intake. For high-risk VVS-CI cases, pacemaker implantation is optional but not universally beneficial [12,24]. Multiple studies have further validated the benign prognosis of HUTT-induced cardioinhibitory VVS. Guaraldi et al. [25] demonstrated that HUTT-triggered cardiac arrest does not predict adverse mortality or major cardiovascular events. Carvalho et al. [26] analyzed a large syncope cohort and found low syncope recurrence and zero mortality after standardized conservative intervention, with pacemaker and drug therapy showing no significant impact on syncope episode frequency [27]. These findings collectively confirm the favorable overall prognosis of pediatric cardioinhibitory VVS.

Currently, high-performance prognostic prediction models for pediatric VVS remain scarce. Clinical predictors of HUTT positivity and syncope recurrence remain controversial. Kazemi et al. [28] demonstrated that conventional demographic and baseline rhythm indicators fail to predict HUTT positivity, while age and presyncope symptoms are weak predictors of hypotensive responses during tilt testing. Long-term follow-up data indicated that most single-episode VVS patients achieve favorable outcomes without pharmacological intervention [27]. Existing pediatric-specific predictive indicators mainly focus on orthostatic training-related acceleration index and HR dynamic changes during presyncope [29,30,31]. However, unified, validated prognostic models for cardioinhibitory VVS are still lacking.

Although the combination of HR0 and HUTT mode provides convenient, low-cost prognostic stratification for pediatric cardioinhibitory VVS with a moderate AUC of 0.710, the predictive power is limited by the absence of refined autonomic quantitative indicators. Future research can integrate autonomic function indices, heart rate variability parameters, somatic reflex sensitivity, and autonomic dysfunction biomarkers to construct a more accurate and robust prognostic model, thereby improving clinical risk stratification and individualized intervention guidance.

This study found that in pediatric VVS exhibiting cardioinhibitory responses induced by HUTT, compared with the good prognosis group, the HR0 in the poor prognosis group was lower and the proportion of SNHUT was higher. This is contrary to the direction of the influence of HR0 and HUTT modes on the syncope episodes in pediatric VVS [32,33,34], which might be related to the fact that this study only included pediatric VVS exhibiting cardioinhibitory responses. The relatively low HR in the baseline state in the poor prognosis group in this study might be related to the increased vagal nerve activity, suggesting that HR0 has an independent protective effect on the prognosis of VVS. The increase in the proportion of SNHUT might be related to the fact that nitroglycerin stimulation is prone to causing autonomic nerve function imbalance, suggesting that SNHUT is an independent risk factor for the prognosis of VVS. Thus, the combination of HR0 and HUTT modes can have a relatively good predictive effect on the prognosis of pediatric VVS exhibiting cardioinhibitory responses induced by HUTT.

## 5. Limitations

This study has several limitations. First, this is a retrospective, observational, and single-center study with a 20-year enrollment time span for study subjects. The long study period and single-center design may introduce bias in key data and limit the generalizability of the findings, as the enrolled population cannot fully represent the clinical scenarios in medical institutions of different regions and tiers. Further multicenter, large-sample prospective studies are required to validate the results before broader extrapolation and clinical application. Second, although nitroglycerin can improve the positive detection rate of HUTT, the specificity of nitroglycerin-assisted testing could not be adequately evaluated due to the lack of relevant data from healthy controls.

## 6. Conclusions

In pediatric VVS exhibiting cardioinhibitory responses during HUTT, the HR0 is significantly lower, and the proportion of SNHUT is significantly higher in the poor prognosis group compared with the good prognosis group. HR0 serves as an independent protective factor, whereas SNHUT functions as an independent risk factor for the prognosis of VVS. The combined assessment of HR0 and HUTT modes demonstrates robust predictive value for the efficacy of non-pharmacological interventions on VVS prognosis.

## Figures and Tables

**Table 1:** Comparison of general data between the good prognosis group and the poor prognosis group ($\bar{x}$ ± *s*, n (%)).

	Good Prognosis	Poor Prognosis	Standardize Diff.	*p*-Value
n	233	170		
Age (year)	11.03 ± 2.53	11.33 ± 2.47	0.12 (−0.08, 0.32)	0.232
Sex			0.04 (−0.16, 0.24)	0.698
Male	101 (43.35)	77 (45.29)		
Female	132 (56.65)	93 (54.71)		
Height (cm)	146.97 ± 15.07	147.79 ± 14.78	0.05 (−0.15, 0.26)	0.595
Weight (kg)	37.62 ± 12.38	38.10 ± 11.02	0.04 (−0.16, 0.24)	0.688
Syncope times (times)	1.95 ± 3.46	1.91 ± 3.19	0.01 (−0.19, 0.22)	0.896
Base line				
HR0 (bpm)	79.17 ± 13.51	75.67 ± 12.21	0.27 (0.07, 0.47)	0.008
SBP0 (mmHg)	106.70 ± 10.82	106.16 ± 9.97	0.05 (−0.15, 0.25)	0.609
DBP0 (mmHg)	65.66 ± 7.57	65.48 ± 7.08	0.02 (−0.17, 0.22)	0.810
Syncopal attack				
HRs (bpm)	80.61 ± 32.16	86.74 ± 32.89	0.19 (−0.01, 0.39)	0.064
SBPs (mmHg)	76.96 ± 16.31	78.83 ± 14.36	0.12 (−0.09, 0.33)	0.256
DBPs (mmHg)	43.07 ± 12.47	43.08 ± 12.86	0.00 (−0.21, 0.21)	0.992
Hemodynamic type			0.24 (0.05, 0.44)	0.060
VVS-VI	149 (63.95)	126 (74.12)		
VVS-CI	13 (5.58)	4 (2.35)		
VVS-M	71 (30.47)	40 (23.53)		
HUTT modes			0.77 (0.56, 0.97)	0.001
BHUT	113 (48.71)	26 (15.29)		
SNHUT	119 (51.29)	144 (84.71)		

HR0, baseline heart rate; SBP0, baseline systolic blood pressure; DBP0, baseline diastolic blood pressure; HRs, syncopal attack heart rate; SBPs, syncopal attack systolic blood pressure; DBPs, syncopal attack diastolic blood pressure; HUTT, head-up tilt test; BHUT, basic head-up tilt test; SNHUT, sublingual nitroglycerin-provoked head-up tilt test; VVS-VI, vasoinhibited type vasovagal syncope; VVS-CI, cardioinhibited type vasovagal syncope; VVS-M, mixed type vasovagal syncope.

**Table 2:** Univariate analysis of VVS prognosis ($\bar{x}$ ± *s*, n (%)).

	Statistics	OR (95%CI)	*p*-Value
Age (years)	11.16 ± 2.51	1.05 (0.97, 1.14)	0.232
Sex			
Male	178 (44.17)	1.0	
Female	225 (55.83)	0.92 (0.62, 1.38)	0.698
Height (cm)	147.32 ± 14.94	1.00 (0.99, 1.02)	0.594
Weight (kg)	37.82 ± 11.82	1.00 (0.99, 1.02)	0.687
Syncope times (times)	1.93 ± 3.34	1.00 (0.94, 1.06)	0.896
Base line			
HR0	77.69 ± 13.08	0.98 (0.96, 0.99)	0.009
SBP0	106.47 ± 10.46	1.00 (0.98, 1.01)	0.608
DBP0	65.59 ± 7.36	1.00 (0.97, 1.02)	0.810
Syncopal attack			
HRs	83.18 ± 32.57	1.01 (1.00, 1.01)	0.065
SBPs	77.77 ± 15.50	1.01 (0.99, 1.02)	0.257
DBPs	43.07 ± 12.63	1.00 (0.98, 1.02)	0.992
Hemodynamic type			
VVS-VI	275 (68.24)	1.0	
VVS-CI	17 (4.22)	0.36 (0.12, 1.14)	0.084
VVS-M	111 (27.54)	0.67 (0.42, 1.05)	0.080
HUTT modes			
BHUT	139 (34.58)	1.0	
SNHUT	263 (65.42)	5.26 (3.22, 8.59)	0.000

HR0, baseline heart rate; SBP0, baseline systolic blood pressure; DBP0, baseline diastolic blood pressure; HRs, syncopal attack heart rate; SBPs, syncopal attack systolic blood pressure; DBPs, syncopal attack diastolic blood pressure; HUTT, head-up tilt test; BHUT, basic head-up tilt test; SNHUT, sublingual nitroglycerin-provoked head-up tilt test; VVS-VI, vasoinhibited type vasovagal syncope; VVS-CI, cardioinhibited type vasovagal syncope; VVS-M, mixed type vasovagal syncope. Result variable: prognosis. Exposed variable: age, sex, height, weight, hemodynamic type, syncope times, HR0, SBP0, DBP0, HRs, SBPs, DBPs, HUTT modes. Adjusted variable: none.

**Table 3:** Prediction models of VVS prognosis evaluated by HR0 and HUTT mode.

	Model I		Model II	
	OR (95%CI)	*p*-Value	OR (95%CI)	*p*-Value
HR0	0.98 (0.96, 0.99)	0.0117	0.97 (0.95, 1.00)	0.0191
HUTT modes				
BHUT	1.0		1.0	
SNHUT	5.75 (3.47, 9.54)	<0.0001	4.34 (2.47, 7.63)	<0.0001

Result variable: prognosis; exposure variable: HR0/HUTT modes; Model I in HR0 adjusted for: age, sex, height, weight. Model II in HR0 adjusted for: age, sex, height, weight, hemodynamic type, syncope times, SBP0, DBP0, HRs, SBPs, DBPs, HUTT modes; Model I in HUTT modes adjusted for: age, sex, height, weight. Model II in HUTT modes adjusted for: age, sex, height, weight, hemodynamic type, syncope times, HR0, SBP0, DBP0, HRs, SBPs, DBPs.

**Table 4:** ROC evaluation of combined indicators (HR0 + HUTT mode) to predict the prognosis of VVS.

Indicator	AUC	95%CI	*p*-Value	Sensitivity (%)	Specificity (%)	Positive Predictive Value	Negative Predictive Value	Jordon Index
HR0 + HUTT modes	0.710	0.660, 0.760	0.000	68.82	65.67	2.00	0.47	0.35

## Data Availability

The datasets used and/or analyzed during the current study are available from the corresponding author on reasonable request.

## Abbreviations

AUC: area under the curve; HR: heart rate; BP: blood pressure; SBP: systolic blood pressure; DBP: diastolic blood pressure; HR0: baseline heart rate; SBP0: baseline systolic blood pressure; DBP0: baseline diastolic blood pressure; HRs: syncopal attack heart rate; SBPs: syncopal attack systolic blood pressure; DBPs: syncopal attack diastolic blood pressure; HUTT: head-up tilt test; BHUT: basic head-up tilt test; SNHUT: sublingual nitroglycerin-provoked head-up tilt test; VVS-VI: vasoinhibited type vasovagal syncope; VVS-CI: cardioinhibited type vasovagal syncope; VVS-M: mixed type vasovagal syncope.
